# Reduced Intensity Conditioning Allogeneic Hematopoietic Stem Cell Transplantation for Acute Lymphoblastic Leukemia; Current Evidence, and Improving Outcomes Going Forward

**DOI:** 10.1007/s11899-018-0462-x

**Published:** 2018-07-14

**Authors:** Jessica T. Leonard, Brandon Hayes-Lattin

**Affiliations:** Center for Hematologic Malignancies, Knight Cancer Institute, Oregon Health and Science University, 3181 SW Sam Jackson Park Rd, Mail Code L586, Portland, OR 97239 USA

**Keywords:** Acute lymphoblastic leukemia (ALL), Allogeneic hematopoietic stem cell transplant (HSCT), Reduced intensity conditioning (RIC)

## Abstract

**Purpose of Review:**

Outcomes for older adults with acute lymphoblastic leukemia (ALL) remain poor, and allogeneic hematopoietic stem cell transplant (HSCT) remains a potentially curative modality. However, benefits are offset by high rates of non-relapse mortality (NRM) in patients undergoing myeloablative conditioning (MAC) regimens. Reduced intensity conditioning (RIC) regimens can extend this therapy to adults who are unfit for MAC, although at the cost of higher relapse rates. In this review, we discuss evidence to support the usage of RIC regimens, controversies, and potential strategies to improve transplant outcomes going forward.

**Recent Findings:**

Several novel therapies have recently been approved for the treatment of relapsed ALL and may play an important role in bridging adults with residual disease to RIC transplant. Assessing response to initial therapy via minimal residual disease (MRD) monitoring may determine which patients will derive the most benefit from allogeneic HSCT.

**Summary:**

Reduced intensity allogeneic HSCT remains a potentially curative therapy that can be offered to older adults however challenges remain. Going forward, MRD testing and novel therapies may help better select which patients should proceed to transplant and assist in getting those patients to transplant with optimally controlled disease.

## Introduction

Outcomes for older adults with acute lymphoblastic leukemia remain poor, with an estimated 5-year survival of approximately 35–45% [1]. This is in part due to the inability of older adults to tolerate the intensive therapies used to treat pediatric and young adult patients [2, 3]. Allogeneic hematopoietic stem cell transplantation (HSCT) has been shown to improve survival in adults with ALL as compared to chemotherapy alone [4, 5]; however, transplant-related mortality (TRM) increases with age. In a study from the CIBMTR, 5-year TRM in adults undergoing myeloablative allogeneic HSCT was reported as ranging between 33 and 58% [6], and is higher for adults with a poor performance status [7]. Reduced intensity conditioning (RIC) offers the chance to extend a potentially curative strategy and graft vs. leukemia effect to older patients without the associated toxicities of a myeloablative regimen. In this article, we will first review the indication for allogeneic hematopoietic transplant in adult ALL, compare outcomes of RIC to MAC conditioning, review issues related to RIC, discuss unanswered questions and controversies in RIC, and discuss potential methods to improve outcomes with RIC. Finally, we will discuss how novel agents recently approved for the treatment of ALL and how they may be combined/sequenced with transplant.

### The Role of Transplant in Adult ALL

The indications for allogeneic hematopoietic stem cell transplant in CR1 are controversial, with recommendations from major groups ranging from transplant for all adults with ALL to recommending this procedure to only those with a matched donor available [4, 8–11]. However, for those who suffer a relapse the long term overall survival is < 24% [5, 12], stressing the need to identify those who will benefit from transplant early in the disease course. A common method is to stratify patients based on risk factors (Table 1), with transplant in CR1 offered to all patients with adverse characteristics. However, more recent evidence suggests that conventional high-risk factors may be trumped by minimal residual disease (MRD) status (to be discussed later). Until prospective studies can clearly define a subset of adults that achieve durable long-term remissions without transplant, transplant in CR 1 is generally recommended for all “adults” with high-risk disease.

**Table 1:** High Risk Features in Acute Lymphoblastic Leukemia

Risk factor	Value
Age	Age > 35* In general, higher risk with increasing age
WBC count	B-lineage: WBC > 30 k T-lineage: WBC > 100 k
Cytogenetics	High risk: t(9;22)** (4;11) t(v; 11q23) any MLL rearrangement t(8;14) t(17;19) Monosomy 7 Low hypodiploidy or near tetraploidy Complex cytogenetics (> 5 chromosomal abnormalities) Intermediate risk: t(1;19) Trisomy 8 or trisomy 21 Deletion 6q Good risk: Hyperdiploidy (51–65 chromosomes) t(12;21)—ETV6-RUNX1
Immunophenotype	Poor prognostic markers: B-lineage: CD20 expression*** T-lineage: ETP-ALL Good prognostic marker: T-lineage: cortical phenotype (CD1a)
Molecular markers	B-lineage: Ph-like ALL (CRLF2, ABL, PDGFR) IKZF1 deletion TP53 mutation
Response to Induction therapy	Failure to achieve hematologic remission within 4 weeks of induction therapy

^T^Adapted from *Hematopoietic Cell Transplants – Concepts, Controversies and Future Directions.* Cambridge University Press May 2017

*Commonly used cut off however with pediatric inspired regimens being used to the age of 39, would consider increase in age to ≥ 40

**Since the development of the ABL specific kinase inhibitors, many report outcomes of Ph+ ALL as similar to those of patients with Ph− ALL

***The poor prognostic impact of CD20 expression may be overcome with addition of Rituximab to standard chemotherapy

### Defining Adult—Where Do We Draw the Line—the Role of Intensive Chemotherapy in ALL

The classification for “high risk” includes age, which is currently defined as over 35 years.

Several large retrospective studies have now demonstrated that adolescent and young adults treated with a pediatric-based regimen have superior outcomes, upward of 60–70% without consolidative allogeneic HSCT [3, 11, 13–15]. Despite the retrospective nature of these studies, the current culture has been to avoid transplant in this age group unless they relapse or are persistently MRD positive. A major question/controversy in this field is what the upper age limit for these regimens should be, as this limit has ranged between 30 and 60 years in different studies. While one group did report that a pediatric-based regimen was tolerated up to the age of 50 [11], other groups have found higher rates of toxicity when these regimens are used in patients over the ages of 35–45 [2, 3]. Many contemporary prospective trials of pediatric-based regimens are limiting the upper age limit to < 40 years (CALGB 10403, Alliance AO41501), and the majority of data regarding the use of pediatric regimens are in adults up to the age of 30–39. To date, there are limited data to suggest that using “adult” regimens results in durable remission without the use of transplant, with the possible exception of MRD-guided strategies (see separate discussion below). For that reason, the current NCCN recommendation is that outside of a clinical trial, all adults who are not treated with a pediatric-based regimen should be considered for allogeneic HSCT in CR1, with the lower age limit being defined by currently available pediatric-inspired protocols.

### MAC vs. RIC

As younger adults may be treated with chemotherapy alone, the transplant-eligible population is thus older and more likely to suffer the side effects of myeloablative conditioning. For this reason, many groups are offering reduced intensity conditioning regimens to their adults who are eligible for transplant. Unfortunately, to date, there have been no prospective studies comparing outcomes of patients with acute leukemias who undergo MAC vs. RIC conditioning regimens to determine whether outcomes are equivalent. However, there are several large retrospective cohort reports that have addressed this question [7, 16–18]. As these studies are the basis for which we justify reduced intensity conditioning, it is worth reviewing them in detail.

In a CIBMTR study, Marks et al. [16] examined the role of the intensity of conditioning regimen on relapse rate, non-relapse mortality (NRM), and overall survival (OS) in adults > 35 years of age with Philadelphia chromosome negative ALL. For definitions of RIC, please see Table 2. The majority of patients who underwent MAC received TBI, generally in combination with cyclophosphamide, and a smaller number had high-dose busulfan-based regimens. Between the years of 1995–2006, a total of 1428 patients who underwent MAC and 93 patients who underwent RIC were identified. As a group, patients who underwent RIC were older (45 vs. 28 years), with 43% of this group being > 50 years of age. Those that were younger who underwent RIC had a KPS < 80, organ dysfunction or a history of invasive fungal infection. Other differences between the two groups included source of graft (more peripheral blood grafts in the RIC group) and more RIC being performed in the recent time period. The authors found that there was no significant difference between the two groups in terms of 3-year NRM (MAC 33%, RIC 32%, *p* = 0.92) and although there was a trend toward higher risk of relapse in the RIC group, this was not statistically significant (26% MAC vs. 35% RIC, *p* = 0.08). The age-adjusted overall survival was also not statistically different (43% MAC vs. 38% RIC, *p* = 0.39), suggesting that RIC is a suitable alternative to MAC for older adults. In a multivariate analysis, factors that negatively impacted overall survival included Karnofsky performance status < 80, mismatched-unrelated donors, transplant in CR2 and age > 30 years.

**Table 2:** Summary of cooperative studies comparing outcomes with reduced intensity conditioning as compared to myeloablative conditioning in adult ALL

Study	Years conducted	Definition reduced intensity	Patient ages	Number of subjects	Patient characteristics	Donor source	Outcomes
Marks et al. CIBMTR	1995–2006	Busulphan 9 mg/kg or less; melphalan 150 mg/kg or less; TBI < 500 cGy single dose or < 800 cGy fractionated; fludarabine + low-dose TBI	MAC: 28 (16–62) RIC: 45 (17–66)	MAC: 1428 RIC: 93	Ph− B-ALL, T-ALL CR1 or CR2	Matched sibling, MUD, MMUD	3-year OS: MAC 43%, RIC 38% *p* = 0.39 NRM: MAC 33%, RIC 32%, *p* = 0.86 Relapse: MAC 26%, RIC 25%, *p* = 0.08)
Tanaka et al. JSHCT	2000–2009	Busulphan 9 mg/kg or less; melphalan 140 mg/kg or less; fludarabine + low-dose TBI	MAC: 51 (45–70) RIC: 58 (45–70)	MAC: 369 RIC: 206	B-ALL, both Ph+ and Ph−, T-ALL CR1 or CR2	Matched sibling, MUD, MMUD	z3-year OS: MAC 51%, RIC 53% *p* = 0.701 NRM: MAC 15%, RIC 26%, *p* = 0.008 Relapse: MAC 31%, RIC 47%, *p* < 0.001
Mohty et al.EBMT	1997–2007	Busulphan 8 mg/kg or less; melphalan 150 mg/kg or less; fludarabine + low-dose TBI	MAC: 50 (45–68) RIC: 56 (45–73)	MAC: 449 RIC: 127	B-ALL, both Ph+ and Ph− CR1 or CR2	All donors were fully matched siblings	2-year OS: MAC 45%, RIC 48% *p* = 0.56 NRM: MAC 31%, RIC 21%, *p* = 0.03 Relapse: MAC 31%, RIC 47%, *p* < 0.001
Bachanova et al. CIBMTR, Ph+ ALL only	2000–2009	Consistent with CIBMTR guidelines above	MAC: 50 (19–66) RIC: 54 (19–69)	MAC: 130 RIC: 67	Ph+ ALL only, all patients in CR1	Matched sibling, MUD, MMUD	3-year OS: MAC 35%, RIC 39% *p* = 0.701 NRM (1-year): MAC 36%, RIC 13%, *p* < 0.001 Relapse: MAC 28%, RIC 49%, *p* < 0.058

*MA*C myeloablative conditioning regimens, *RIC* reduced intensity conditioning regimens, *ALL* acute lymphoblastic leukemia

Similar results were seen in a cooperative group study from the JSHCT [18]. The impact of conditioning regimen on adults > 45 years of age with both Ph+ and Ph− ALL were examined. Between the years of 2000–2009, 575 patients (369 MAC, 206 RIC) were identified who met inclusion criteria. There was no statistically significant difference in the 3-year rate of NRM (38% MAC vs. 36% RIC); however, there was a higher risk of relapse in the RIC group which did not become apparent until the third year after transplant (risk of relapse at 1 year 12% MAC vs. 14% RIC; risk of relapse at 3 years 15% MAC vs. 26% RIC, *p* = 0.008). As above in a multivariate analysis, there was no significant effect of conditioning intensity on 3-year overall survival (51% MAC vs. 53% RIC, *p* = 0.701). Factors associated with a worse outcome in this group included HLA-mismatched donors and transplant in CR2.

While both of these studies suggest that outcomes with RIC are similar to outcomes for MAC in adults, results should be interpreted with caution. The patient populations were heterogeneous in terms of donor source and GVHD regimens, and although criteria for the definition of RIC were applied, a wide variety of conditioning regimens were used. In addition, differences between the groups (older age and more comorbidities in the RIC groups, more common use of PBSC source and more recent transplant) make drawing conclusions difficult.

Two additional studies are worth discussion, as they used more uniform populations. Mohty et al. [7] on behalf of the EBMT described the effect of conditioning regimen on outcomes in adults > 45 years of age with both Ph+ as well as Ph− acute lymphoblastic leukemia. This study was limited to patients who underwent a matched sibling allogeneic stem cell transplant in a complete remission (CR1 or CR2). A total of 576 patients met the inclusion criteria between the years of 1997–2007, of these 449 underwent MAC and 127 underwent RIC. The median age of the RIC group was 56 (45–73) and that of the MAC group was 50 (45–68). Other significant differences included age of donor (49 years in the MAC group and 55 in the RIC group, *p* < 0.001), bone marrow source (34% of MAC patients vs. 9% of RIC patients), and female donor into male recipient (22%of MAC vs. 31% RIC, *p* = 0.05). In contrast to the study by Marks et al., the authors found that 2-year transplant-related mortality was significantly reduced in the RIC group (29% MAC vs. 21% RIC, *p* = 0.03); however, the risk of relapse was significantly higher (31% MAC vs. 47% RIC, *p* < 0.001). However, this translated into no significant difference in overall survival between the two groups, with the 2-year OS rate being 45% in the MAC group and 48% in the RIC group (*p* = 0.56).

One additional study focused on the effect of conditioning regimen on Ph+ patients alone. Bachanova et al. [17••] on behalf of the CIBMTR compared outcomes of 197 adults ages > 18 who underwent MAC (130) vs. RIC (67) in first CR. In their analysis, patients were matched for age, donor type, and HCT year. The majority of patients received pre-transplant TKI, which was similar between the groups. Similar to the study by Mohty, TRM was found to be significantly lower in the RIC when compared to the MAC group (36% MAC vs. 13% RIC, *p* = 0.001). Additionally, rates of aGVHD were significantly lower in the RIC group (30 vs. 47%, *p* = 0.014). However, the 3-year relapse rate was higher in the RIC group (49 vs. 28%, *p* = 0.058). Factors associated with a higher risk of relapse in the RIC group included MRD positivity as well as no pre-HCT TKI. Consistent with all other studies, 3-year overall survival was not significantly different between the two groups (39% RIC vs. 35% MAC).

Although all groups reported no significant difference in overall survival between myeloablative conditioning vs. reduced intensity conditioning, it is interesting to note that in two of the studies, the differences between rates of TRM and relapse between the myeloablative and RIC groups was more apparent. The major difference in these studies is that some variables were more uniform, with patients in the Mohty study being limited to those who received sibling donors and patients in the Bachanova study being limited to those in CR1. In addition, in the Bachanova study, patients in the RIC vs. MAC group were further matched in terms of donor source, age, and comorbidities. This highlights the challenges with interpreting results from large retrospective cohorts, as the heterogeneity of patient populations, donor source, and disease status can confound any true differences between conditioning regimen intensity. Although a randomized trial comparing RIC to MAC is unlikely to occur, a large, well-designed prospective study of RIC allogeneic HSCT in older adults with ALL would help delineate the risks and benefits of this transplant strategy.

### Single Institution Studies of RIC

A number of groups have published single institution studies reporting their outcomes with reduced intensity conditioning [19–23] (Table 3). Many of these include adults with both AML and ALL with only a small number of ALL patients, or look at a variety of conditioning regimens, making results difficult to interpret. However, two are worthy of discussion. In a study from the City of Hope, 24 patients aged 23–68 years underwent fludarabine/melphalan conditioning for high-risk ALL defined as either over age 50, compromised organ function, or prior HSCT. Forty-two percent of the patients were Philadelphia chromosome positive (Ph+) and three patients had ALL that was secondary to a prior malignancy. They reported an impressive 2-year OS of 61.5%, with a 2-year NRM of 21.5%, and a relapse incidence of 21.1% [19]. In another study, a Korean group reported the outcomes of 37 adults aged 15–63 with high-risk ALL who underwent FluMel conditioning. Patients were defined as high risk if they were > 50 years of age, had compromised organ function, or active infection that would preclude them from undergoing myeloablative conditioning. Only patients in CR1 and CR2 were included in this study. This group reported similarly impressive outcomes, with a 3-year OS rate of 64%, 3-year NRM of 17.7%, and 3-year relapse rate of 19.7% [20]. These outcomes are significantly better than those reported by the large cooperative group studies above, and are likely due to the fact that these studies were performed at single institutions, where there was more uniformity in terms of not only conditioning regimen, but also GVHD prophylaxis and supportive care. This suggests that if conditions can be optimized and patients properly selected, outcomes for adults with RIC can approach those of the AYA population treated with a pediatric regimen.

**Table 3:** Single Institution Studies of reduced intensity conditioning (RIC) regimens in adult ALL

Study	Regimen	Years conducted	Patient ages	Number of subjects	Disease status	Donor source	Outcomes
Stein et al	FluMel	2002–2007	23–68	24	Ph+/Ph− CR1: 11 CR2: 4 CR ≥ 3: 4 Relapsed: 4 Refractory: 1	Sibling: 8 MUD: 8 MMUD: 6	2 year: OS 61.5% NRM: 21.5% Relapse: 21.1%
Cho et al	FluMel	2000–2007	15–63	37	Ph+/Ph−T-cell CR1: 30 CR2: 7	Sibling: 27 MUD: 4 MMUD: 6	3-year: OS: 64.1% NRM: 19.7% Relapse: 17.7%
Ram et al	Flu/TBI	2000–2009	8–69	51	Ph+/Ph− CR1: 25 CR1, MRD+: 7 CR ≥ 2: 18 Persistent disease: 1	Sibling: 9 MUD: 31 MMUD: 11`	3-year: OS: 34% NRM: 28% Relapse: 40%
Hamaki et al	Flu/Bu Flu/Bu + ATG Flu/Bu + TBI Flu/Bu + ATG + TBI Flu/Mel Cladribine-based Other	2000–2003	17–68	33	CR1: 13 CR2: 6 Relapsed: 9 Refractory: 5	Sibling: 20 MM sib: 5 Unrelated: 8	3-year: progression: 50.9% progression-free mortality: 30.4% 2-year: OS: 29.7%
Massenkeil et al.........	Flu/Bu + ATG	1998–2002	19–67	9	Unknown for ALL subset	Unknown for ALL subset	3-year: OS: 44%

### Disease Status at Time of Transplant—Who may Not Benefit From RIC Transplant?

A consistent finding across many of the studies reviewed is that disease status at the time of RIC transplant significantly impacts transplant outcomes. The 3-year relapse rates for patients transplanted in ≥ CR2 or with relapsed/refractory disease have been reported to be as high as 67%, and 3-year overall survival is reported as being 20% or less. This still offers an improvement in survival as compared to those who do not undergo allogeneic HSCT after relapse, where survival is < 10%. However, the benefit is modest and a thorough discussion of transplant associated morbidity should be had with patients prior to deciding whether to proceed with transplant at later disease states. One important factor impacting survival in relapsed patients is the presence of active disease; for those who proceed to transplant with disease present survival is < 10%, with some groups reporting no long-term survivors for patients who proceed to transplant with active disease [12, 21]. Although myeloablative regimens may offer a chance for survival for these patients [24], reduced intensity transplant should not be recommended to patients with active disease.

### Age vs. Performance Status, Which Should Limit Who Is Eligible for Transplant?

Many of the cooperative group studies discussed above define adult as patients over age 18, but what about outcomes when we focus on older adults only? A retrospective CIBMTR study examined the outcomes of adults over 55 years of age who underwent reduced intensity conditioning between the years of 2001–2012 [25••]. Two hundred seventy-three patients were identified, ranging in age between 55 and 72. The majority of the patients were in CR1 (71%), with 17% being in CR2 or beyond and 11% with primary induction failure vs. relapse. Donor source included unrelated donors (59%) vs. a matched sibling (32%). Fifty percent of patients had Ph+ disease, and 56% had a KPS of > 90%. Conditioning regimens varied but were consistent with the previously described CIBMTR definition of reduced intensity. For the group as a whole, the 3-year OS was 38% with a 3-year NRM rate of 25%. Relapse was the leading cause of death in the group, with 47% of all patients suffering from relapse. When stratified by age (55–60, 61–65 and 66+), those 66+ had a higher rate of NRM (40 vs. 22–23% for the younger groups, *p* = 0.07), as well as increased mortality (RR 1.51, 95% CI 1.0–2.29, *p* = 0.05). However, those 66+ also tended to have a worse KPS (≤ 80) and more comorbidities, and KPS ≤ 80 independently was significantly associated with an increased 3-year NRM risk (34%, 95% CI 25–43%). This makes it difficult to discern whether age or performance status contributes more to poor outcomes. Other groups that included younger adults have similarly noted that poor performance status is associated with higher NRM and decreased OS [6, 7], highlighting the fact that performance status in and of itself is indeed a risk for poor outcomes. While there are certainly older adults who proceed to RIC transplant with an excellent performance status and tolerate the procedure quite well, age may still modify the impact of poor performance status, with older individuals with a poor KPS being less able to tolerate transplant than younger patients with a poor KPS. Therefore, transplant, even reduced intensity, should be undertaken with caution in older patients with concurrent comorbidities and poor performance status.

### Questions to Address Going Forward—What Is the Optimal Conditioning Regimen?

One criticism of reduced intensity conditioning is that many regimens do not include TBI, which is thought to reduce the risk of CNS and sanctuary site relapse [26]. In the CIBMTR review comparing MAC to RIC, the addition of TBI did significantly lower the risk of relapse; however, this included both patients undergoing MAC and RIC, in which the majority of patients that had received TBI were MAC recipients [16]. In addition, the Fred Hutchison group that reported outcomes of adults treated with Flu/TBI reported that no patients suffered from isolated CNS relapse [21]. When confined to just RIC regimens, the EBMT group reported no differences in NRM or rate of relapse when comparing low-dose TBI-based regimens to fludarabine-busulphan to fludarabine-melphalan, respectively (NRM 18 vs. 23 vs. 23%, *p* = NS and relapse 48 vs. 55 vs. 45%, *p* = NS). Absent from all group studies was reports of CNS/sanctuary site relapse vs. marrow relapse, and there have been no prospective trials directly comparing TBI-based to non-TBI-based RIC regimens. Therefore, the role of TBI in the context of reduced intensity regimens remains to be defined.

Another unanswered question concerning reduced intensity regimens is how reduced can the intensity be? In a six-institution study coordinated by Fred Hutchison cancer center, adults ages 8–69 years with high-risk ALL underwent conditioning with a non-myeloablative regimen that included fludarabine 90 mg/m^2^ and 2Gy TBI [21]. Patients were defined as high risk if they were over the age of 50–55, had prior myeloablative allogeneic HSCT, or had other comorbidities that precluded the use of a myeloablative regimen. Both Ph+ as well as Ph− patients were included, and disease status at the time of transplant ranged from CR1 to >CR3 and patients with relapsed/refractory disease. The 3-year OS in this group was reported as 34%, with a 3-year NRM of 28% and a 3-year relapse rate of 40%. However, when limited to patients transplanted in CR1, the 3-year OS rate was 62%. NRM was not described for the group of patients who underwent transplant in CR1; however, the 3-year rate of relapse in this group was between 15% (Ph−) and 32% (Ph+). Although comparisons between studies is challenging given the differences in patient populations, it is encouraging to see that outcomes from a non-myeloablative conditioning regimen were on par with those from single-institution RIC studies for patients in CR1.

### Donor and Graft Source, Does it Matter? Enhancing GVL

Given the higher reliance on the graft vs. leukemia (GVL) effect in patients undergoing reduced intensity conditioning prior to allogeneic HSCT, the source of the graft has the potential to significantly impact the risk of disease relapse. Early transplant studies suggested that the best donor source for patients with ALL was a matched sibling, and many trials that stratified to transplant vs. no transplant based on donor availability only considered matched siblings. However, with improvements in supportive care and higher level HLA matching, outcomes for adults with ALL who undergo an 8/8 matched unrelated donor transplant are equivalent to that of patients who undergo a matched sibling transplant [27–30]. Rates of both acute as well as chronic GVHD were higher in the MUD group; however, relapse rates were significantly lower, presumably due to a more potent graft vs. leukemia effect. It is possible that in the reduced intensity setting where more of the therapeutic effect is based on graft vs. leukemia, an unrelated donor may provide an advantage over a matched related donor. Similar findings were seen in a single institution study from Dana Farber, which included patients with any hematologic malignancy, where outcomes from adults who underwent reduced intensity conditioning and received a transplant from a matched sibling were compared to outcomes for those who underwent compared outcomes for adults with a variety of hematologic malignancies, who underwent a matched unrelated donor transplant [28]. Patients who underwent MUD transplant had lower rates of relapse at 2 years (52 vs. 65%, p = 0.005) and superior 2-year PFS (39.5 vs. 29%, p = 0.01). Overall survival at 2 years was not significantly different between the groups, being 56% for MUD vs. 50% for MRD (p = 0.53). Only seven patients with ALL were included in this study, so it is difficult to say whether these findings would be pertinent to patients with ALL. In addition, given the morbidity associated with GVHD, it is premature to recommend that patients who are undergoing RIC should seek out an unrelated donor. However, going forward as we learn more about the biology of GVL vs. GVHD and have better methodologies to prevent and treat GVHD, the current paradigm of preferring a matched sibling donor may come into question, particularly in the context of a reduced intensity conditioning regimen.

Peripheral blood stem cells (PBSC) vs. bone marrow (BM) as a donor source is another factor that may impact outcomes after reduced intensity conditioning. In a retrospective analysis from the EBMT, outcomes for adult patients with AML who underwent reduced intensity conditioning and received either PBSC or BM from either matched related vs. matched unrelated were compared. In the context of receiving a matched sibling donor graft, there was no difference in GVHD or leukemia free survival between patients receiving PBSC vs. BM grafts. However, for patients who underwent MUD transplant, the group that received PBSC had higher rates of both acute and chronic GVHD as well as higher NRM, but also had significantly lower incidence of relapse [29]. This is in contrast to a retrospective study published by the CIBMTR, where no difference in rates of NRM, relapse or overall survival were found between patients with hematologic malignancies that underwent PBSC vs. BM transplant [30]. In the CIBMTR analysis, however, both related and unrelated donors were analyzed together. Neither of these studies included patients with ALL, and thus these findings cannot be generalized to this population. At the present time, the majority of adults undergoing allogeneic HSCT in the USA receive PBSC grafts given the ease of collection, although using BM as a source is more common in Europe. As we develop better treatments for GVHD and are able to lower rates of NRM, PBSC may become the preferred source for patients undergoing RIC conditioning as a way to enhance GVL.

### Strategies to Improve RIC—Pre-transplant MRD and Post-transplant Maintenance

Minimal residual disease (MRD) has been shown to be a strong predictor of outcome after allogeneic HSCT, with several groups reporting inferior outcomes for patients that enter transplant with MRD of > 10^–4^ [31–35]. MRD status would be expected to be even more important in the context of reduced intensity conditioning, where the therapeutic benefit is derived from the graft vs. leukemia effect rather than by cytoreduction through a myeloablative conditioning. This was demonstrated in the CIMBTR study of reduced intensity conditioning in patients with Ph+ ALL, in which patients who were MRD positive pre-transplant had a higher risk of relapse if they underwent RIC vs. MAC conditioning [17••] (61% RIC vs. 35% MAC, HR 1.97 with 95% CI 1.09–3.57, *p* = 0.026). Although this direct comparison between MRD positivity and risk of relapse in RIC vs. MAC conditioning has not been studied in Ph− ALL, it is clear that MRD is a marker for worse outcomes after transplant. However, there are many unanswered questions concerning MRD. First, it is unclear what the optimal time to determine MRD status, with some groups reporting the significance of early MRD clearance [36, 37•], while others report that early MRD positivity (at week 6) is not as predictive of post-transplant outcome as MRD level after consolidation at week 16 and/or week 22 [38]. Next, the optimal threshold for determining what level of MRD positivity is relevant has not yet been determined. Current protocols define MRD as anything > 10^–4^; however, with the advent of commercially available next-generation sequencing (NGS) for all subtypes of ALL through IG and TCR sequencing, it is possible to detect MRD to less than 1 × 10^–7^. It is not yet clear whether more sensitivity is better, or whether very low-level disease can still be overcome by even reduced intensity conditioning regimens. Finally, although blinatumomab was recently shown to improve outcomes when used to treat patients with MRD positivity (see next section), the number of patients treated is small and it remains to be see whether treating a patient who is MRD positive to a deeper level of remission prior to transplant will improves outcomes in the context of reduced intensity conditioning. 

An alternate method to improve transplant outcomes is the use of post-transplant maintenance therapy for high-risk patients. This has already proven to be beneficial for patients with Ph+ ALL who are treated with post-transplant TKIs [39–41], and some groups have reported that pre-transplant MRD levels are no longer significant in this patient population [42]. Whether post-transplant TKI therapy should be administered prophylactically to all patients undergoing allogeneic HSCT for Ph+ ALL vs. just for those who develop MRD after transplant (i.e., a pre-emptive strategy) is an open area of debate, although one small prospective study did show equivalent outcomes for patients who received prophylactic vs pre-emptive therapy [40]. Although there are currently no accepted post-transplant therapies for Ph− ALL or T-ALL, post-transplant maintenance therapy is an active area of investigation and is a potential strategy to mitigate the high-risk status of patients entering a RIC transplant with MRD.

### Novel Therapies

Treatment options for patients with relapsed ALL have greatly increased in the recent years, with the FDA approval of three new agents in this setting. All therapies are highly effective, and will thus allow more patients with relapsed disease to attain disease control and proceed to a potentially curative allogeneic HSCT. In addition, when effective, all of therapies have reported high rates of MRD negativity, and thus could serve as a useful bridge to allogeneic HSCT.

Blinatumomab is a bispecific T cell engaging antibody (BiTE) composed of an anti-CD19 antibody connected to an anti-CD3 antibody via a non-immunogenic link. It functions by bringing cytotoxic T cells in contact with CD19+ cells, leading to perforin-mediated lysis of leukemic cells. A randomized phase III trial of blinatumomab compared to salvage chemotherapy for patients with relapsed/refractory ALL showed that 44% of patients in the blinatumomab arm attained a CR, CRh, or Cri within the first 12 weeks as compared to 25% in the standard chemotherapy group [43•]. Seventy-six percent of the responders in the blinatumomab group achieved a MRD-negative status, as compared to 48% of responders in the chemotherapy group. Response was correlated to percentage of blasts present in the bone marrow, with 65.5% of patients with < 50% bone marrow blasts responding to treatment as compared to 34.4% of those with > 50% marrow blasts. Although shown to be superior to chemotherapy, the real benefit of blinatumomab may be in treating patients with low levels of persistent disease to an MRD-negative state. An early pilot study of blinatumomab looked at response rate in patients with ALL who had persistent MRD after initial therapy or those who became MRD positive after initially attaining molecular remission [44]. Sixteen of 20 patients (80%) became MRD negative after 1 cycle, and long-term follow-up showed that 33 months and then 5 years, 60 and 50%, or patients remained in remission, respectively [45]. Based on these promising results, a recent phase II study investigated the use of blinatumomab in patients in a hematologic CR (< 5% blasts) but with persistent MRD positivity (> 1 × 10-3). Both patients in CR1 but with persistent MRD as well as patients in CR 2-3 with persistent MRD after salvage therapy were included. After a single cycle of blinatumomab, 78% of patients became MRD negative, and in a landmark analysis responders had a significantly longer relapse free survival (23.6 vs 5.7) and OS (38.9 vs 12.5 months) as compared to the non-responders. Seventy four patients underwent allogeneic hsct; of these 49% remain in remission, 15% have relapsed and 18% died in CR. [46••]. Although the benefit of treating MRD pre-transplant to improve RFS and OS has not yet been demonstrated in a randomized trial, blinatumomab has now become the first agent approved to address MRD positivity. Ongoing trials incorporating blinatumomab into the in post-transplant setting to prevent MRD emergence are ongoing; results will help define the optimal usage of blinatumomab as it relates to allogeneic HSCT.

Inotuzumab ogozamycin is a drug-antibody conjugate that combines the toxin calicheamicin with a humanized CD22 antibody. When this antibody binds to B cells, it is rapidly internalized, delivering the drug directly to the leukemic blasts. A phase III trial comparing salvage chemotherapy to treatment with inotuzumab also showed promising results, with an overall response rate (CR + CHR + Cri) of 81% as compared to 29% in the salvage chemotherapy arm. Of the responders in the inotuzumab group, 78% achieved a MRD negative status [47•]. In addition, a greater proportion of patients was able to proceed to allogeneic HSCT in the inotuzumab arm; 41 vs. 11%. In regard to transplant, however, inotuzumab carries a risk of developing veno-occlusive disease (VOD) for patients who had previously or who subsequently underwent allogeneic HSCT. The overall risk of VOD for patients treated with inotuzumab was 11%, however for transplant patients 10/48 developed VOD. The risk appears to be higher for patients who underwent conditioning with a dual-alkylator regimen. As there is a newly approved agent available for the treatment of VOD (defibrotide) not all cases were fatal; 2 patients recovered, 4 had ongoing VOD, and 1 died. As the majority of RIC regimens contain only one alkylating agent if any, the hope would be that rates of VOD would be small or negligible in this population. However, this remains to be determined.

Finally, one of the most exciting new strategies for treating relapsed/refractory ALL is genetically engineered T cells, the chimeric antigen receptor T cells (CAR-T cells). In this therapy, T cells are harvested from a patient and transduced with the receptor for CD19; the T cells are then able to exert a cytotoxic effect on cells that highly express CD19 (i.e., leukemic blasts). Cells are also transduced with a co-stimulatory domain, either 4-1BB or CD28; these “second generation” CAR-T cells show enhanced efficacy and longer duration of persistence after infusion. Responses to CAR-T cells are impressive, with rates ranging between 70 and 94% in very heavily pre-treated patients [48–52]. As with both inotuzumab and blinatumomab, among patients who responded, the majority, > 80%, attained a MRD-negative status. CAR-T cell therapy does come with a high rate of toxicity, including cytokine release syndrome and neurotoxicity, which has led to a number of deaths. While FDA approved for patients under the age of 25, results in adults have not been as impressive, as the toxicities associated with CAR-T cells are not as well tolerated in older individuals [53, 54]. In addition to toxicity, relapse is another issue with CAR-T cells, with the primary mechanism being relapse with CD19 negative disease followed by immunologic response to the cells [48, 55, 56]. Although many hope that CAR-T cell therapy will ultimately replace allogeneic HSCT, much work remains to be done to enhance durability and better understand and manage the serious toxicities associated with this therapy.

Although each of these novel agents represents an important step forward for the treatment of patients with relapsed/refractory B-ALL, few durable remissions without consolidative allogeneic HSCT have been reported. Until we have a better understanding of which patients may achieve a long-term remission, and until we can prolong the durability of CAR-T cells, these therapies remain a bridge to allogeneic HSCT.

### Moving Away From Transplant (for Some), MRD-Guided Strategies

As mentioned previously, MRD remains a strong predictor of outcome in acute lymphoblastic leukemia. Within the pediatric realm, MRD-directed therapy is increasingly being used to intensify or de-escalate therapy [57]. This begs the question, can MRD-directed therapy be used to identify a subset of adults for whom allogeneic HSCT is not necessary?

Several large cooperative group studies have attempted an MRD-guided approach in adults, with promising results. In three separate studies, the Italian NILG trial, the German GMALL 07/2003 trial, and the Spanish PETHEMA trial, adult patients were treated with a pediatric inspired regimen and then assigned to undergo consolidation with chemotherapy if they were MRD negative or allogeneic HSCT if they were MRD positive. Although each group used a different method to measure MRD as well as a different time point to assess MRD, all identified a subset of low-risk, MRD-negative patients with relapse rates of < 20–30% without allogeneic HSCT [5, 31, 39]. However, whether these results can be applied to older adults is not yet clear. There were few adults above the age of 50–55 in these trials, and presumably the promising outcomes in the chemotherapy only group are in part secondary to the pediatric based regimens, which are not as well tolerated in older adults. There are no data at this time to suggest that “adult” regimens can produce durable remissions without consolidative allogeneic HSCT. In addition, 20–30% of MRD “negative” patients do still relapse, and survival for adults transplanted in CR2 is substantially lower than for those transplanted in CR1 [24]. As we learn more about MRD with time, we may be able to identify a subset of older adults for whom transplant is not necessary. However, the ideal “adult” treatment regimen that will provide outcomes equivalent to those of pediatric-inspired regimens remains to be determined. For now, transplant in CR1 for older adults remains the standard of care as by definition, all have “high-risk” disease.

## Conclusion

Acute Lymphoblastic leukemia has a bimodal age distribution, with an increased incidence not only in childhood but also in adults > 50. An estimated 28% of ALL patients are over the age of 45, and thus represent a substantial number of yearly cases. While the introduction of pediatric-based treatment regimens has greatly improved outcomes for adolescents and young adults, likely obviating the need for allogeneic HSCT for many, the toxicities of these regimens increases substantially over the age of 40–50. For older adult patients, the benefit of myeloablative allogeneic HSCT is outweighed by the risks; however, reduced intensity conditioning is a valid option for these patients. Therefore, more effort should be made toward optimizing outcomes in the reduced intensity setting. This could include incorporating novel agents into upfront treatment of adult ALL to increase rates of MRD negativity prior to transplant, optimizing conditioning regimens to reduce TRM, optimizing donor selection to enhance the GVL effect, and using prophylactic or pre-emptive post-transplant therapies.
